# Potential Role of Captive Environments in Reshaping the Compositions of Pathogenic Gut Bacteria in *Equus* Species

**DOI:** 10.3390/biology15100796

**Published:** 2026-05-16

**Authors:** Haotian Li, Xinyuan Hou, Shile Han, Songtao Xie, Yuchun Li, Xibao Wang

**Affiliations:** 1 College of Agriculture and Biology, Liaocheng University, Liaocheng 252059, China; lihaotian@lcu.edu.cn (H.L.);; 2 Marine College, Shandong University (Weihai), Weihai 264209, China; li_yuchun@sdu.edu.cn; 3 College of Life Sciences, Qufu Normal University, Qufu 273165, China

**Keywords:** *Equus* species, pathogenic bacteria, 16S rRNA gene, wild and captive environment

## Abstract

Captive environments can have detrimental effects on the health of *Equus* species. However, due to the limitations of available databases, the types and abundance of pathogenic bacteria in *Equus* species remain largely unknown. Therefore, this study aimed to explore pathogenic gut bacteria in wild and captive *Equus* species. Using 16S rRNA gene sequencing and a comprehensive multiple bacterial pathogen detection database, we compared the pathogenic gut bacterial profiles of three *Equus* species (*Equus ferus przewalskii*, *E. hemionus*, and *E. kiang*) across different captive and wild environments. The pheatmap revealed that the three *Equus* species living in different captive environments showed convergence in their pathogenic gut bacterial composition. Captive *Equus* species had significantly enriched zoonotic bacteria, whereas wild *Equus* species had significantly enriched animal pathogenic bacteria. These findings suggest that a captive environment may increase the risk of zoonotic bacterial transmission between *Equus* species and humans. We hope that this study can provide a reasonable scientific basis for the management and protection of captive *Equus* species.

## Simple Summary

*Equus ferus przewalskii*, *E. hemionus*, and *E. kiang* are classified as first-class protected animals (Category I) in China. Ex situ breeding is one of the primary strategies for the conservation and recovery of these three *Equus* species. This study analyzed gut pathogens in wild and captive *Equus* species. We found that the relative abundance of zoonotic pathogens was significantly higher in captive individuals than in wild ones. Thus, captive environments may increase the risk of *Equus* species contracting zoonotic diseases. Therefore, we hope this study contributes to improving the health management of captive *Equus* species.

## 1. Introduction

The gut microbiome plays a pivotal role in host immunity and metabolic homeostasis across mammalian species [1,2]. In endangered wildlife conservation, microbial dysbiosis induced by captivity has been increasingly recognized as a critical factor affecting animal health [3]. Three species of the wild *Equus* (*Equus ferus przewalskii*, *E. hemionus*, and *E. kiang*) are found in China [4]. They are listed as a First-Grade State Protection animals in China, with the *E. f. przewalskii* listed as endangered by the International Union for Conservation of Nature, under criteria D [5]. Currently, captive breeding is one of the main methods to protect and rescue wild *Equus* species [6,7]. However, a captive environment can lead to unfavorable conditions such as sub-health and disease in *Equus* species [8]. This may be associated with an increased abundance of pathogenic bacteria in the gut microbiome of *Equus* species [8,9]. Recent studies have demonstrated that captivity-induced gut microbiome dysbiosis is associated with increased pathogen loads and disease susceptibility in endangered mammals. For instance, captive skywalker hoolock gibbon (*Hoolock tianxing*) and black rhinoceros (*Diceros bicornis*) exhibit higher abundance of pathogens such as Spirochetes [10] and *Bacteroides fragilis* [11]. These findings underscore the need to investigate how captive environments reshape pathogenic microbial communities in *Equus* species.

The gut microbiomes of *Equus* species are complex micro-ecosystems that contain bacteria (probiotics and pathogenic bacteria, etc.), fungi, and viruses [7,12,13,14,15,16]. Probiotic bacteria can provide energy and maintain health in these species [17]. In contrast, an increased abundance of pathogenic bacteria can cause diseases and pathological processes such as inflammation [14,18,19,20,21]. Therefore, researchers have explored the health of captive *Equus* species through gut microbiomics [6,14]. Based on the Greengenes and Silva databases, many studies have found that the abundance of potentially pathogenic bacteria is increased in the gut microbiome of captive *Equus* species [6,21,22,23]. Although the Greengenes and Silva databases provide descriptions of gut bacterial composition, a significant portion (13–15%) of the *Equus* gut microbiome remains unclassified. Moreover, pathogenic bacteria are not defined or classified in the Greengenes and Silva databases. As a result, the types and abundances of pathogenic bacteria in *Equus* species have not been characterized in detail.

We hypothesized that captive *Equus* species harbor higher abundance of zoonotic pathogens compared to wild counterparts, driven by human-associated microbial transmission. Thus, this study aimed to examine and compare the pathogenic gut bacteria in wild and captive members of the genus *Equus*. We analyzed 96 fecal samples from three *Equus* species across wild and captive environments, integrating alpha/beta diversity and taxonomic composition to analyze the abundance composition of zoonotic pathogens in captive *Equus* species. Our results indicate that the captive environment increases the abundance of zoonotic pathogenic bacteria in captive *Equus* species.

## 2. Materials and Methods

### 2.1. Sample Collection

Data were downloaded from the National Center for Biotechnology Information (NCBI) and selected based on the following criteria: (1) The data source includes *Equus* species from both wild and captive environments; (2) Sequencing samples were derived from fecal material; (3) The host was a healthy individual; (4) The sequencing region targeted the V3–V4 hypervariable regions of the 16S rRNA gene; (5) All fecal samples were collected in sterile centrifuge tubes, transported in mobile refrigerator at −20 °C during transportation, and stored below −20 °C until sequencing; and (6) The number of data points for each wild and captive environment exceeds 5 to ensure statistically significant.

After screening, a total of 96 samples were used for analysis [7,21,24,25,26]. Information on these samples is listed in Table S1. According to species and survival environment, we designated *E. f. przewalskii* (EFEP), *E. hemionus* (EHEM), and *E. kiang* (EKIA) in the wild environment as WEFEP, WEHEM, WEKIA_1, and WEKIA_2 (WEFEP and WEHEM, 44°36′ N, 88°30 E; WEKIA_1, 35°46.77′ N, 95°15.15′ E; WEKIA_2, 38°.37′ N, 101°.06′ E), respectively. Those in the captive environment were designated as CEFEP_1 (Gansu endangered animals protection center), CEFEP_2 (Xinjiang uygur autonomous region wild horse breeding research center), CEHEM (Gansu endangered animals protection center), CEKIA_1 (Jinan zoo), CEKIA_2 (Jinan wildlife world), and CEKIA_3 (Qinghai-Xizang plateau wild animal park), respectively.

### 2.2. Data Analysis

The primers and barcodes were removed from the raw reads using EasyAmplicon software V1.18.1 [27]. Clean reads were used for pathogenic gut bacterial analysis based on the Multiple Bacterial Pathogen Detection (MBPD) database [28]. For sequence clustering, the amplicon sequence variant (ASV) approach was employed, and species annotations were aligned to the MBPD reference database (18 August 2022) using the UCLUST algorithm, with a confidence threshold of 0.8 [28]. The relative abundance of pathogenic bacteria at various taxonomic levels (phylum to species) was normalized using the EasyAmplicon software V1.18.1 [27]. Alpha diversity indices (richness and Shannon) and beta diversity indices (Manhattan distance) were calculated using the EasyAmplicon software V1.18.1 [27]. Alpha diversity was visualized with a boxplot (Wilcoxon test, *p* < 0.05) by cloudtutu platform (https://cloudtutu.com (accessed on 1 March 2026)), and the rarefaction curves (richness diversity) and heatmap (Manhattan distances) were plotted using sp-peatmap script in EasyAmplicon software V1.18.1 [27]. The types of pathogenic bacteria in wild and captive *Equus* species were compared using the Kruskal–Wallis test (*p* < 0.05), visualized through cloudtutu platform (https://cloudtutu.com (accessed on 7 March 2026)). At the species level, we used the ‘ggplot2’ package in R software (V4.2.1) to generate relative abundance bar cumulative plots of the top 10 bacteria. Additionally, the Linear discriminant analysis Effect Size software (LEfSe, V1.1.2; LDA score > 3, *p* < 0.05) was employed to detect significantly different pathogenic gut bacteria between wild and captive *Equus* species.

## 3. Results

### 3.1. Overview of the Data and Diversity Analysis

After quality control, we obtained 6,131,161 effective reads from 96 samples, with an average of 63,866.26 effective reads per sample. A total of 7136 ASVs were identified in the 96 samples. The rarefaction curves (richness diversity) approached a plateau, indicating that the sequencing depth was sufficient for experimental analysis (Figure S1).

**Figure 1:**
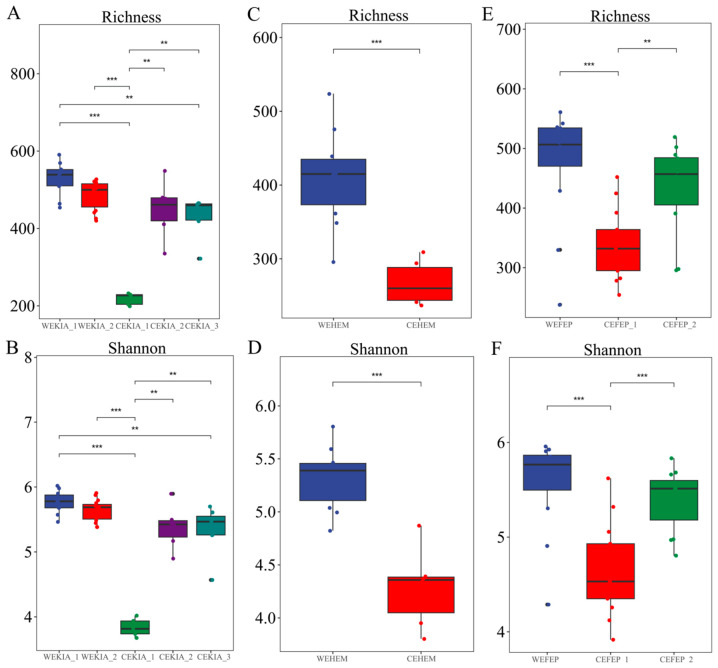
Boxplot of Alpha diversity (Wilcoxon’s test). (**A**) Difference in richness diversity between WEKIA (1–2) and CEKIA (1–3); (**B**) difference in Shannon diversity between WEKIA (1–2) and CEKIA (1–3); (**C**) difference in richness diversity between WEHEM and CEHEM; (**D**) difference in Shannon diversity between WEHEM and CEHEM; (**E**) difference in Richness diversity between WEFEP and CEFEP (1–2); (**F**) difference in Shannon diversity between WEFEP and CEFEP (1–2). ** *p* < 0.01, *** *p* < 0.001. N_WEKIA_1_ = 10, N_WEKIA_2_ = 15, N_WEHEM_ = 10, N_WEFEP_ = 12, N_CEKIA_1_ = 5, N_CEKIA_2_ = 6, N_CEKIA_3_ = 7, N_CEHEM_ = 6, N_CEFEP_1_ = 13, N_CEFEP_2_ = 12.

The results of the Wilcoxon tests for richness and Shannon indices are shown in a boxplot (Figure 1). The diversity and richness of pathogenic gut bacteria in wild *Equus* species were higher than those in captive *Equus* species. In different captive environments, the gut pathogenic bacteria of the same species exhibit varying alpha diversity. For example, group CEKIA_2 and CEKIA_3 were significantly higher than CEKIA_1, while the group CEFEP_2 was significantly higher than the group CEFEP_1. These findings suggest that *Equus* species are exposed to a greater variety of pathogenic bacteria in the wild environment. The composition of different environmental factors (such as environmental microbiome and diets) in different captive environments may significantly affect the diversity of gut pathogenic bacteria in *Equus* species.

### 3.2. Cluster and Pathogenic Bacteria Type Analyses

**Figure 2:**
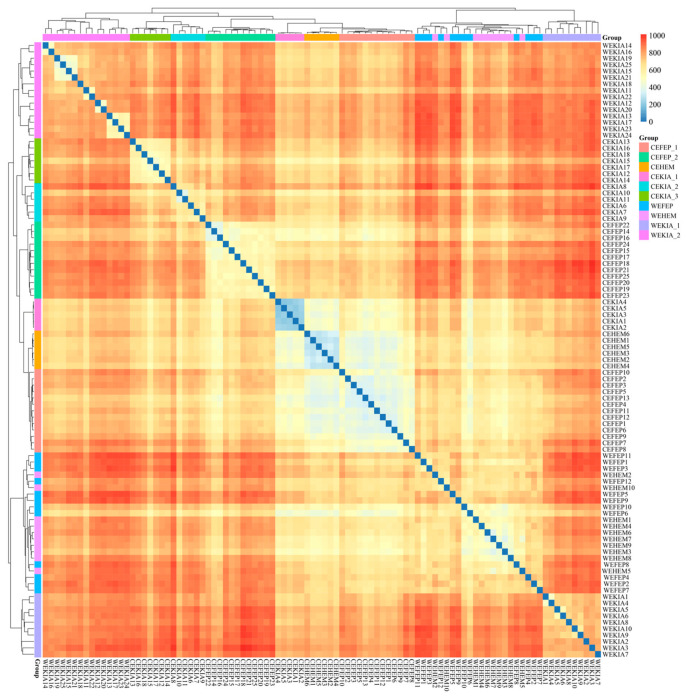
Pheatmap plot of gut pathogenic bacteria composition of all samples. A darker red color indicates a greater distance between two samples (i.e., the value approaches 1), while a lighter red color suggests a smaller distance between the two samples (i.e., the value approaches 0). N_WEKIA_1_ = 10, N_WEKIA_2_ = 15, N_WEHEM_ = 10, N_WEFEP_ = 12, N_CEKIA_1_ = 5, N_CEKIA_2_ = 6, N_CEKIA_3_ = 7, N_CEHEM_ = 6, N_CEFEP_1_ = 13, N_CEFEP_2_ = 12.

A heatmap was used to show the differences in pathogenic gut bacterial composition between wild and captive *Equus* species (Figure 2). The captive *Equus* species (CEKIA_1, CEKIA_2, CEKIA_3, CEHEM, CEFEP_1, and CEFEP_2) formed a separate cluster. Except for WEKIA_2, WEKIA_1, WEHEM, and WEFEP clustered together. Although the samples of group CEFEP_2 were sampled in winter, their pathogenic bacterial composition was similar to that of individuals sampled in spring and summer. Furthermore, the pathogenic gut bacterial composition of the three *Equus* species was similar in the five captive environments. We hypothesized that the same pathogenic bacteria may be present in different captive environments, resulting in a similar composition of pathogenic gut bacteria in captive *Equus* species.

**Figure 3:**
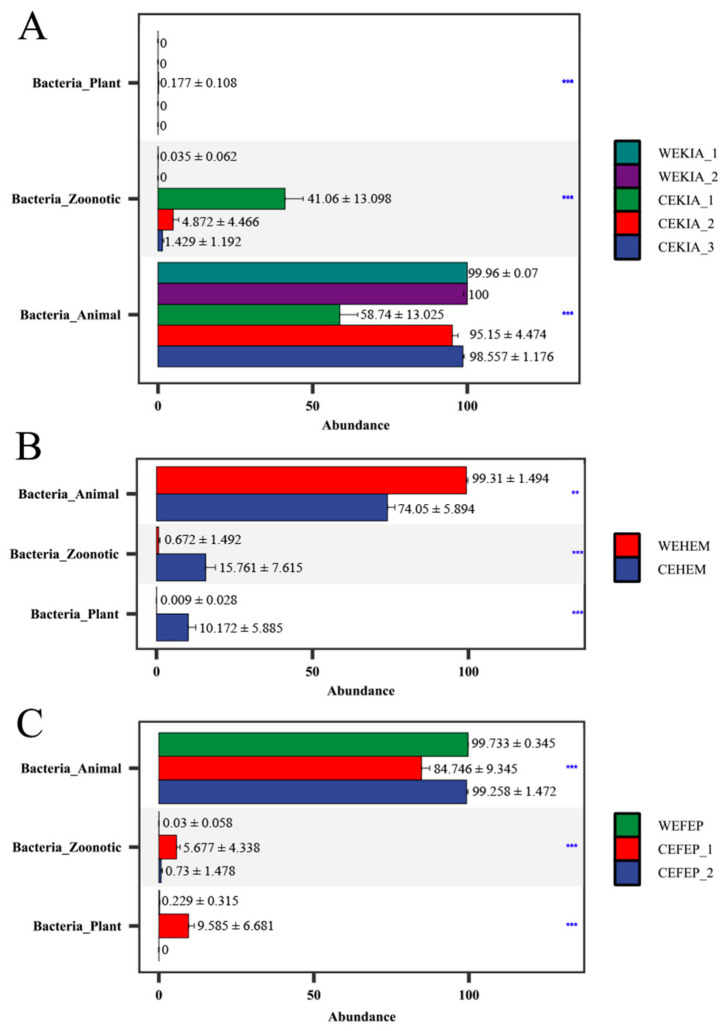
Kruskal–Wallis test for pathogenic bacteria type between wild and captive *Equus* species ((**A**) captive and wild *E. kiang*; (**B**) captive and wild *E. hemionus*; (**C**) captive and wild *E. f. przewalskii*). ** *p* < 0.01, *** *p* < 0.001. N_WEKIA_1_ = 10, N_WEKIA_2_ = 15, N_WEHEM_ = 10, N_WEFEP_ = 12, N_CEKIA_1_ = 5, N_CEKIA_2_ = 6, N_CEKIA_3_ = 7, N_CEHEM_ = 6, N_CEFEP_1_ = 13, N_CEFEP_2_ = 12.

Based on the pathogenic bacteria type analysis (Figure 3), we observed that the relative abundance of zoonotic bacteria was significantly higher in captive *Equus* species (CEKIA_1, CEKIA_2, CEKIA_3, CEFEP_1, CEFEP_2, and CEHEM) than in wild *Equus* species (WEKIA_1, WEKIA_2, WEFEP, and WEHEM). Conversely, the relative abundance of animal pathogenic bacteria was significantly higher in wild *Equus* species (*p* < 0.05). The sum of average relative abundances of zoonotic and plant pathogenic bacteria in captive individuals (12.41%) was significantly higher than that in wild individuals (0.22%). Based on the results of Figure 2 and Figure 3, we merged CPKIA (1–3), CEFEP (1–2), and WEKIA (1–2) into CPKIA, CEFEP, and WEKIA, respectively.

### 3.3. Pathogenic Gut Bacteria Compositions

The unidentified bacteria and top 10 species occupied more than 80% and 16% of all samples, respectively. The average total abundance of the other 45 bacterial species does not exceed 4% of the gut pathogenic bacteria composition. Therefore, we used cumulative plots of relative abundance for the top 10 bacteria to further analyze the composition of pathogenic gut bacterial communities in wild and captive *Equus* species.

**Figure 4:**
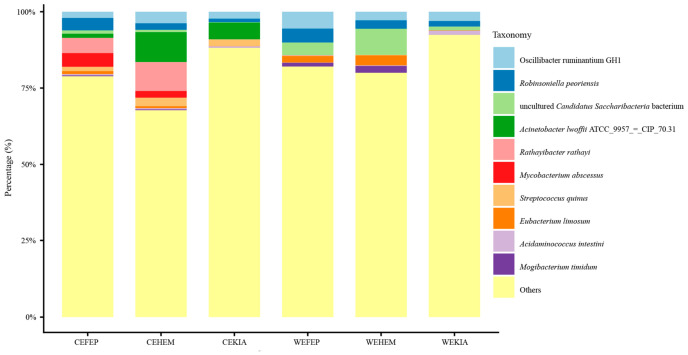
Gut pathogenic bacteria composition of captive and wild *Equus* species at the species level. N_WEKIA_ = 25, N_WEHEM_ = 10, N_WEFEP_ = 12, N_CEKIA_ = 18, N_CEHEM_ = 6, N_CEFEP_ = 25.

As shown in Figure 4, the most abundant species in specific wild and captive *Equus* groups are as follows: *Rathayibacter rathayi* (pathogenic type, plant) was observed in groups CEFEP (4.96%); *Acinetobacter lwoffii* ATCC 9957 = CIP70.31 (pathogenic type, zoonotic) was identified in Group CEKIA (5.47%) and CEHEM (9.48%); An uncultured *Candidatus Saccharibacteria* bacterium (pathogenic type, animal) was found in group WEHEM (8.59%); *Oscillibacter ruminantium* GH1 (pathogenic type, animal) was present in group WEKIA (3.06%) and WEFEP (5.52%).

**Figure 5:**
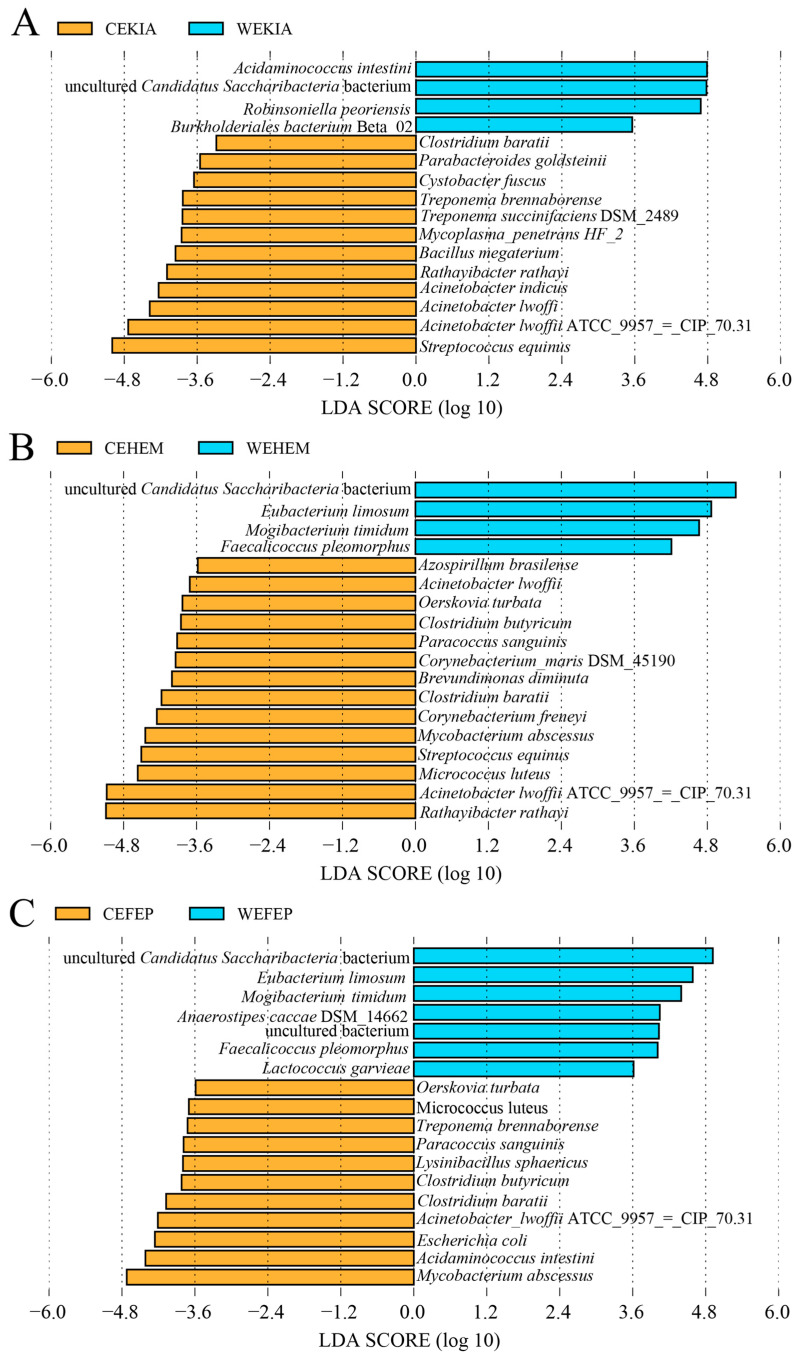
At the species level, LEfSe (LDA Effect Size) analysis between captive and wild *Equus* species ((**A**) captive and wild *E. kiang*; (**B**) captive and wild *E. hemionus*; (**C**) captive and wild *E. f. przewalskii*; LDA > 3, *p* < 0.05). N_WEKIA_ = 25, N_WEHEM_ = 10, N_WEFEP_ = 12, N_CEKIA_ = 18, N_CEHEM_ = 6, N_CEFEP_ = 25.

We performed LEfSe analysis (Figure 5) to identify indicator pathogenic gut bacteria that differ between captive and wild *Equus* species (groups WEKIA and CEKIA; groups WEHEM and CEHEM; and groups CEFEP and WEFEP). Compared to wild *Equus* species, the abundance of some bacterial species only significantly increased in two captive horse species, such as *A. lwoffii* (CEKIA and CEHEM). At the species level, *A. lwoffii* ATCC 9957 = CIP 70.31 and *Clostridium butyricum* were significantly enriched in captive *Equus* species. Uncultured *C. Saccharibacteria* bacterium were more abundant in wild *Equus* species than in captive *Equus* species. Compared to wild *Equus* species, the abundance of some bacterial species only significantly increased in two captive horse species, such as *A. lwoffii* (CEKIA and CEHEM). Similarly, the abundance of *Eubacterium limosum* only significantly increased in WEFEP and WEHEM.

## 4. Discussion

By comparing 16S rRNA data for captive and wild *Equus* species, we found that captive environments reshape the composition of pathogenic gut bacteria. Captive *Equus* species have similar gut pathogen structures, and the abundance of zoonotic pathogenic bacteria is significantly higher than that of wild *Equus* species. The substantial increase in the relative abundance of zoonotic pathogenic bacteria might contribute significantly to the observed similarity in pathogenic bacterial composition between captive *Equus* species. The findings of previous research are consistent with our results. For example, compared to their wild counterparts, captive animals such as *Acinonyx jubatus* [29], *Ursus arctos* [30], and *Peromyscus maniculatus* [31] exhibit higher relative abundances of animal pathogenic bacteria.

In addition, the gut microbiomes of captive *Equus* species have the same indicator pathogens, for example, *A. lwoffii* and *A. lwoffii* ATCC 9957 = CIP 70.31 (genus *Acinetobacter*). Numerous studies have demonstrated that the genus *Acinetobacter* is a zoonotic and opportunistic pathogen commonly found in farms, companion animals, and other environments [32,33,34]. *Acinetobacter* species can cause various diseases, including ventilator-associated pneumonia, bloodstream infections, and meningitis [35]. *Acinetobacter* carries antibiotic-resistant genes, which facilitate its spread between humans and animals [36,37]. Consequently, *Acinetobacter* may represent a significant health risk to individuals who interact closely with animals, including caregivers, zoo visitors, and captive *Equus* species. In the functional prediction, we also found that the abundance of human diseases was significantly higher in captive *Equus* species than in wild *Equus* species. Therefore, captive environments might increase the probability of disease development in *Equus* species [9].

In contrast, animal pathogenic bacteria exhibit higher abundance in wild *Equus* species [31]. For example, *Eubacterium limosum* (naturally present in soil) may facilitate the metabolic conversion of norepinephrine to 3,4-DHPG, resulting in a proconvulsant effect [38,39]. Uncultured *Candidatus Saccharibacteria* bacterium is also often present in natural environments such as soil and water [39]. Therefore, the natural environments represent a significant source of pathogenic gut bacteria in wild *Equus* species.

However, details regarding the welfare, living conditions, and health status of captive *Equus* species, as well as their interactions with other animals and humans, remain unclear. In addition, the age and gender information of some captive animals is unclear. All these factors may influence the abundance and composition of pathogenic bacteria; for example, captive *Equus* species may be in a state of subclinical infection. Although we have confirmed that the captive environment increases the abundance of zoonotic pathogens in *Equus* species, it is currently unclear which specific factors influence the composition of pathogenic bacteria in captive *Equus* species. Therefore, in future studies, we need to collect new samples to clarify the aforementioned information. Through in-depth analysis using multi-omics approaches (metagenomics and metabolomics) in conjunction with factors (such as animal health phenotypes and dietary habits), we aim to elucidate the mechanisms by which the captive environment increases the abundance of zoonotic pathogens in captive *Equus* species.

## 5. Conclusions

We characterized the diversity and composition of pathogenic gut bacteria in captive and wild *Equus* species via 16S rRNA gene sequencing. A captive environment influences *Equus* species to have a more similar composition of pathogenic gut bacterial communities, potentially increasing the probability of zoonotic disease development in these species. Therefore, before entering the enclosure, breeders should wear masks and disinfect their clothes. In addition, breeders must regularly disinfect and sterilize the enclosures of *Equus* species, and may use appropriate amounts of fluoroquinolone antibiotics if necessary. We hope that this study can provide a scientific basis for the protection and management of captive *Equus* species.

## Data Availability

All 16S rRNA gene data was sourced from the National Center for Biotechnology Information (https://www.ncbi.nlm.nih.gov/bioproject/PRJNA701711 (accessed on 20 February 2026); https://www.ncbi.nlm.nih.gov/bioproject/PRJNA837737; https://www.ncbi.nlm.nih.gov/sra/?term=PRJNA553267 (accessed on 20 February 2026); https://www.ncbi.nlm.nih.gov/bioproject/PRJNA558670 (accessed on 20 February 2026); https://www.ncbi.nlm.nih.gov/bioproject/PRJNA436598 (accessed on 20 February 2026)). The code used to analyze was sourced from GitHub (https://github.com/LorMeBioAI/MBPD (accessed on 21 February 2026); https://github.com/YongxinLiu/EasyAmplicon (accessed on 21 February 2026)).

## Supplementary Materials

The following supporting information can be downloaded at: https://www.mdpi.com/article/10.3390/biology15100796/s1, Figure S1: The results of sparse curves (richness diversity); Table S1: The information of the samples used in this study.
