# Supplementary figures and images for: Potential Role of Captive Environments in Reshaping the Compositions of Pathogenic Gut Bacteria in Equus Species
Source: Biology (Basel). 2026 May 16;15(10):796. doi: 10.3390/biology15100796 (PMC13203567; doi:10.3390/biology15100796)

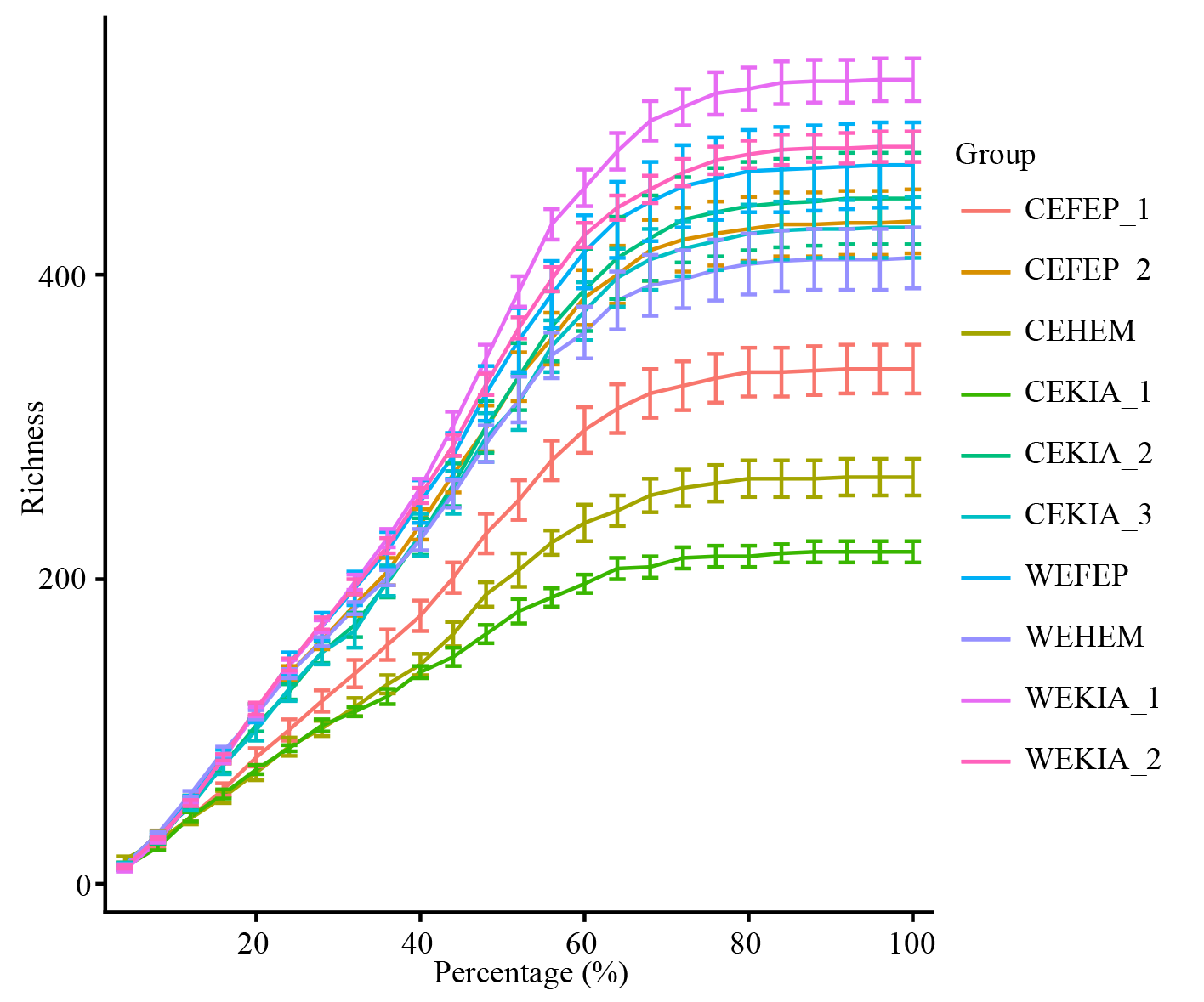

Supplement: Supplementary file 1 [file biology-15-00796-s001.zip › Figure S1.tif]
